# Circulating Fluidized Bed Fly Ash Mixed Functional Cementitious Materials: Shrinkage Compensation of f-CaO, Autoclaved Hydration Characteristics and Environmental Performance

**DOI:** 10.3390/ma14206004

**Published:** 2021-10-12

**Authors:** Wei Zhang, Xiaoming Liu, Zengqi Zhang, Yaguang Wang, Yang Xue, Xiansheng Hao, Yang Lu

**Affiliations:** 1 State Key Laboratory of Advanced Metallurgy, University of Science and Technology Beijing, Beijing 100083, China; zhangwei202101@163.com; 2 School of Metallurgical and Ecological Engineering, University of Science and Technology Beijing, Beijing 100083, China; wangyg@xs.ustb.edu.cn (Y.W.); cdxueyang@163.com (Y.X.); s20190216@xs.ustb.edu.cn (X.H.); luyang1253@163.com (Y.L.)

**Keywords:** circulating fluidized bed fly ash, shrinkage compensation of f-CaO, autoclaved hydration, volume stability, environmental performance

## Abstract

Circulating fluidized bed (CFB) fly ash is a by-product from CFB power generation, which is hard to utilize in cement because it contains f-CaO and SO_3_. This work aims to explore the mechanism of the shrinkage compensation of free-CaO (f-CaO) and the autoclaved hydration characteristics and environmental performance of CFB fly ash mixed cementitious materials (CMM). In this work, long-term volume stability of CMM is improved with the addition of CFBFA. These findings suggest that the compressive strength of sample CMM0.5 is the highest under both standard condition (67.21 MPa) and autoclaved condition (89.56 MPa). Meanwhile, the expansion rate (0.0207%) of sample CMM0.5 is the lowest, which proves the shrinkage compensation effect of f-CaO in CFBFA. The main hydration products of CMM0.5 are Ca_2_SiO_4_•H_2_O (C-S-H) gel, CaAl_2_Si_2_O_7_(OH)_2_•H_2_O (C-A-S-H) gel and Ca(OH)_2_. In addition, the high polymerization degree of [Si(Al)O_4_] and the densified microstructure are presented at the sample CMM0.5. The leaching results indicates that the heavy metals in CMM0.5 satisfies the WHO standards for drinking water due to physical encapsulation and charge balance. Therefore, this investigation provides a novel method of using CFB fly ash in cement.

## 1. Introduction

Circulating fluidized bed (CFB) fly ash is a new type of industrial by-product from power plant, which is different from traditional fly ash [1]. The pozzolonic reactivity of CFBFA is higher than that of coal-powder boiler fly ash, and the SO_2_ produced from low-grade coal could be solidified into CFB fly ash. These characteristics make CFBFA has important environmental, economic and social benefits [2,3,4]. CFB fly ash is produced by CFB boiler [5]. The burning temperature of CFB boiler is 850~900 °C, which is lower than that of coal-powder boiler (1200~1400 °C), indicating that CFB technology is energy saving [6,7]. In addition, the SO_2_ produced by low grade coal is solidified by lime to form CFB fly ash [8], which improves the utilization rate of low-grade coal [9,10,11]. However, the annual production of CFB fly ash is gradually increased with the rapid development of CFB technology. The annual production of CFB fly ash has reached about 65 million tons until 2019 [12]. The storage of CFBFA not only occupies a geat deal of land space, but also pollutes groundwater due to the leaching of the heavy metals [13,14,15,16]. The policies related to utilization of industrial solid waste have been put forward by various countries in recent years [17,18,19]. Therefore, the importance of using CFB fly ash has risen to a new height.

The utilization of CFB fly ash to prepare cement has been widely investigated. Zhou [20] compared the hydration characteristics of CFB fly ash and coal-powder boiler fly ash in cement. The results indicated that the pozzolonic reactivity of CFB fly ash (101.55%) is higher than that of coal-powder boiler fly ash (95.86%) in 90 days, and the densification of hydration products microstructure in CFB fly ash cement is better. Li [21] adopted the way of jet mill to improve the pozzolonicity index (113%) and self-hardening strength of CFB fly ash in 28 days. The CFB fly ash cement with excellent mechanical properties was obtained. Siddique [22] investigated the performance of CFB fly ash cement. The results indicated that the hydration reactivity of cement is activated by pozzolonicity of CFB fly ash, and the water absorption and total porosity of cement are reduced, which leads to the compressive strength of CFB fly ash cement reaching 70 MPa in 91 days. Zahedi [23] explored the influence of pozzolonicity of CFB fly ash on the physical properties and durability of cement. The results indicated that cement mixed with 20 wt.% CFB fly ash obtained the ideal strength and volume stability at 60 days. Jang [24] utilized CFB fly ash to prepare cement strength controllable material, which showed that the engineering and environmental performance of the cement strength controllable material prepared by CFB fly ash meet the ACI 299R-13 in 91 days.

Previous researchers have made great achievements to the improvement of cement strength by introducing active silica–alumina component in CFB fly ash, but the hydration time of CMM in these investigations was mainly concentrated within the initial 3 months. Chen [25] discussed the influence of CFB fly ash on the expansion characteristics of cement paste. The results indicated that the expansion of cement is gradually increased with the content of CFB fly ash from 0 to 80 wt.% at 180 days due to the action of f-CaO. Although the expansion properties of CFB fly ash and the volume shrinkage cracking of cement have been proven in recent years, the shrinkage compensation of cement by f-CaO has not been systematically studied.

The ordinary Portland cement (OPC) and CFB fly ash are compounded in proportion, and the CMM are cured for an appropriate time in the moist cabinet and autoclaved equipment, respectively. The autoclaved equipment was used to increase the hydration reaction rate of CMM to investigate the long-term volume stability of CMM for 360 days. Then, f-CaO shrinkage compensation and autoclaved hydration characteristics of CMM were analyzed via TAM-AIR, XRD, SEM-EDX and ^29^Si MAS NMR, and environmental performance of CMM0.5 was investigated through EPMA and ICP-MS. Therefore, the new method of CFB fly ash application in cement materials is provided by this technology.

## 2. Materials and Methods

### 2.1. Materials

The main raw materials of this experiment are CFB fly ash and P• I 42.5 OPC. P• I 42.5 OPC comes from a cement factory in Hebei Province, China. CFB fly ash is supplied by a power plant in Yangquan City, Shanxi Province, China. The chemical components of the raw materials were determined by XRF (Shimazu Company, Kyoto, Japan), and the results are presented in Table 1. The CFB fly ash contains 39.90 wt.% SiO_2_ and 28.09 wt.% Al_2_O_3_, which contributes to the further development of the CMM strength by C-A-S-H gel. Compared with coal-powder boiler fly ash, the CFB fly ash contains 7.73 wt.% SO_3_ and 4.57 wt.% free-CaO (f-CaO), which is conducive to improving the strength and volume shrinkage compensation of CMM. CFB fly ash contains unburned carbon due to the low burning temperature (850~900 ℃) of CFB boiler. Hence, the ignition loss is 10.38 wt.%. The water absorption of CMM was enhanced with the increase of CFB fly ash content. Therefore, performance of CMM mortar may be deteriorated with the addition of CFB fly ash based on water/cement ratio of 0.5.

**Figure 1:**
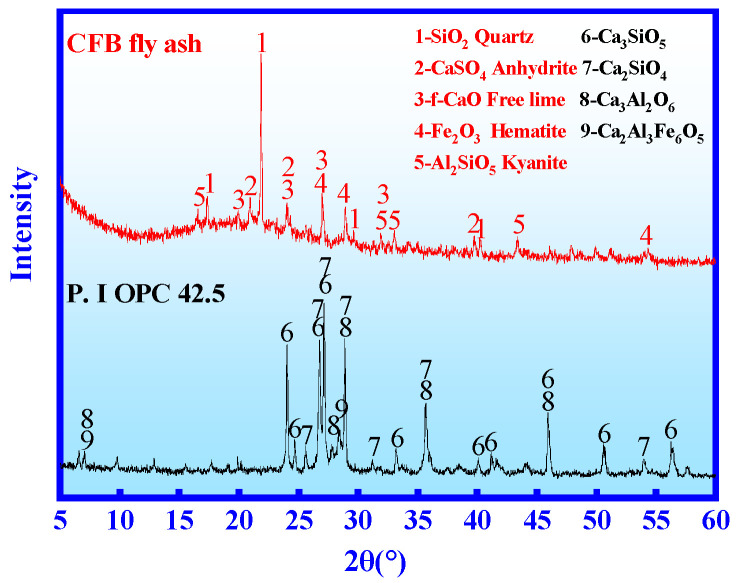
Mineral compositions of CFB fly ash and OPC.

The XRD of CFB fly ash and OPC is provided in Figure 1. The main phase of CFB fly ash are SiO_2_, f-CaO, CaSO_4_, Fe_2_O_3_ and Al_2_SiO_5_. CFB fly ash contains many glassy amorphous phases. The main minerals in OPC are Ca_3_SiO_5_, Ca_2_SiO_4_, Ca_3_Al_2_O_6_ and Ca_2_Al_3_Fe_6_O_5_.

**Figure 2:**
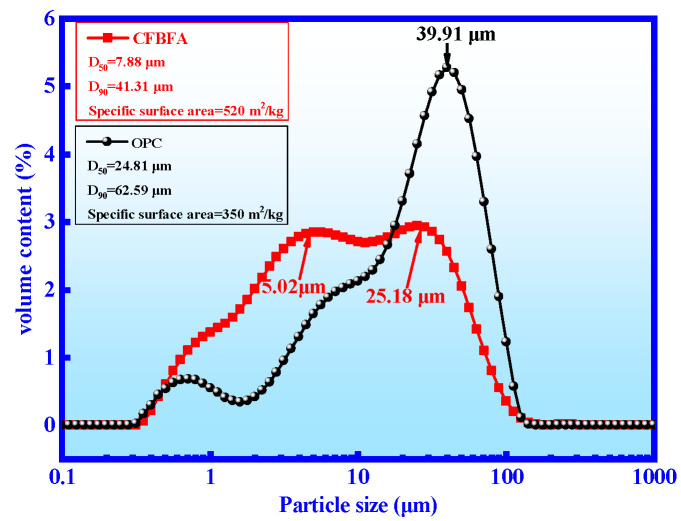
Particle size distribution of CFB fly ash and OPC.

The particle size distribution is an important parameter of raw material. Figure 2 shows the particle size distributions of CFB fly ash and OPC. The particle size range of CFB fly ash is broad, mainly around 5.02 and 25.18 μm, whereas the particle size of OPC is focused on 39.91 μm. The D_50_ and D_90_ of CFB fly ash are 7.88 and 41.31 μm, which are lower than the those of OPC (24.81 and 62.59 μm. The densities of CFB fly ash and OPC are 2375.44 kg/m^3^ and 3062.51 kg/m^3^, respectively. According to GB/T 8074–2008 [26], the specific surface area of CFB fly ash and OPC is 520 m^2^/kg and 350 m^2^/kg, respectively. In additional, the specific surface area of CFB fly ash in this work is higher than that of Zhang (270 m^2^/kg) [1] and Zhou (121 m^2^/kg) [20]. The particle morphology and pore structure of the CFB fly ash are optimized. Therefore, this CFB fly ash could provide better pozzolanic activity and strength for CMM [21].

**Figure 3:**
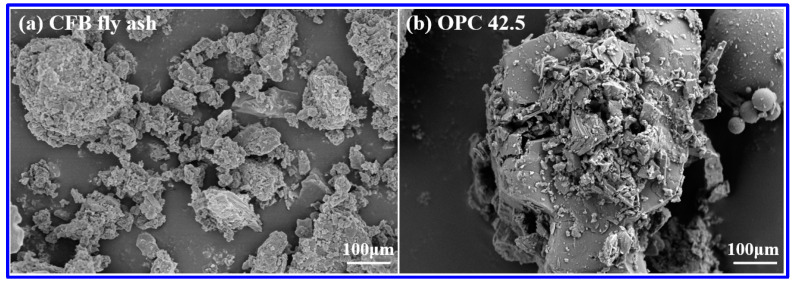
Microscopic morphology of (**a**) CFB fly ash and (**b**) OPC 42.5.

The microscopic morphology of CFB fly ash and OPC are provided in Figure 3. The shape of CFB fly ash particles is non-uniform. Meanwhile, many of the particles are aggregated, and the appearance of the particles possesses many distinct pores. However, the OPC particles is relatively loose, and small particles are exposed on the surface of OPC matrix, which is helpful to hydration reaction.

### 2.2. Proportional Design of CMM

The original CFB fly ash is dried at 105 °C for 24 h. Then, the CFB fly ash is ground on a mechanical grinding machine for 15 min. CMM are prepared by CFB fly ash and OPC. Seven groups of CMM with various mass ratios of T-CaO/(SiO_2_+Al_2_O_3_) (Ca/(Si+Al)) were prepared. As shown in Table 2, the design of CMM0~ CMM5, and the corresponding chemical components is shown in Table 3. It is apparent from Table 2 and Table 3 that the mass ratio of Ca/(Si+Al) decreases from 2.38 to 0.83 with the increase of CFB fly ash from 0 to 50 wt.%, while f-CaO mass ratio increases gradually.

### 2.3. Preparation of CMM

**Figure 4:**
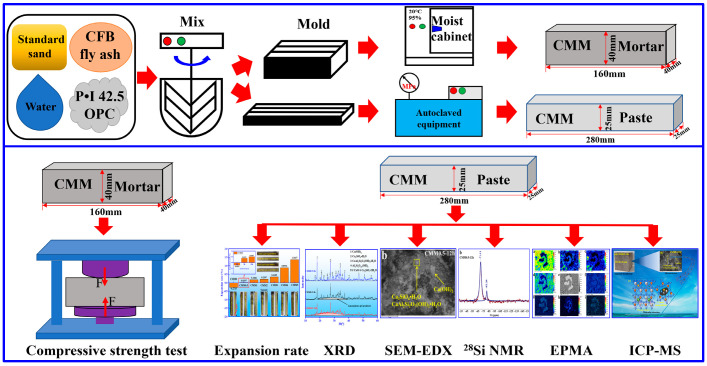
Schematic diagram of CMM preparation process and detection method.

The schematic diagram of preparation and test method of CMM is rendered in Figure 4. The mass percentage of raw material in Table 2 are used to prepare mortar specimens with a dimension of 40 × 40 × 160 mm and a water/cement ratio of 0.5. Then, CMM mortars were cured for 24 h in a moist cabinet (20 ± 1 °C) with relative humidity of 95 ± 1%. In addition, the CMM mortar was removed from the mold and cured in the isothermal moist cabinet at the relative humidity of 95 ± 1% and temperature of 20 ± 1 °C until a certain time. Afterwards, the strength of CMM mortars were examined according to the Chinese standard GB/T 17671-1999 [27]. CMM paste was prepared and molded at 25 × 25 × 280 mm, and it was cured under the same curing conditions as the mortar. The autoclaved test of CMM paste was conducted according to the Chinese standard GB/T 750-1992 [28]. The autoclaved expansion rate of paste was calculated according to Equation (1), and three specimens of each CMM paste were averaged.

(1) $$L_{A}=\frac{L_{1}{-L}_{0}}{L_{0}}\times 100$$

where $L_{A}$ corresponds to autoclaved expansion rate of paste, %; $L_{0}$ represents the initial length of paste after separation from the mold, mm; $L_{1}$ represents the autoclaved length of the paste, mm. The CMM paste is qualified when its expansion rate is less than 0.5%. The reaction of the paste after autoclaved cured for a certain time was terminated by alcohol immersion, and then dried in a vacuum oven at 60 °C for 24 h. The paste was then used for microstructure characterization.

### 2.4. Test Methods

According to the requirements of GB/T17671-1999 [27], the compressive strength of CMM mortar was detected using the Mechanical Strength Tester HYE-300-10 (Beijing Seyu LuTong Instrument Mile Co., Ltd., Beijing, China). The performance results of mortar/paste mix at different hydration ages were the average values of three mortars/pastes. The dispersion of a data set is reflected by standard deviation. The dispersion coefficients corresponding to the properties of mortar/paste are calculated via standard deviation formula. CMM mortar and paste are cured using a cement autoclaved machine YZF-2S (Tianjin ShouKe Experimental Machine Co., Ltd., Tianjin, China). The main autoclaved test parameters were as follows: pressure is 2.0 MPa; temperature was 215.7 °C; the heating rate was 3~4 °C/min [28]. The expansion rate of CMM paste was measured via high precision length measuring instrument based on GB/T 750-1992 [28]. In addition, the mass fraction of f-CaO in CFB fly ash and CMM was detected according to the China standard YB/T 4328-2012 [29].

The exothermic rate and cumulative hydration heat of CMM paste were examined using the isothermal instrument at 20 °C for 72 h. The mineral composition of CMM was examined through X-Ray Powder Diffraction (XRD) of Japan Science and Technology Instrument Co., Ltd. (Kyoto, Japan) The SEM-EDX of CMM was tested using a Gemini 300 thermal Field emission scanning electron microscope and Oxford X-MAX Energy Dispersive X-ray Detector (Hitachi Corporation, Ibaraki, Japan). The ^29^Si MAS spectra of CMM were detected through the JMM-EC600R MAS NMR spectrometer. The distributions of Ca, Al, Si, O, As, Cr, Zn and Cu in the CMM0.5–12 h were examined by the Electro-Probe Microanalyzer (EPMA- JXA-8530F Plus, Kyoto City, Japan) to obtain the qualitative and quantitative results for the above elements. According to GB 5086.1-1997 standard [30], the solid (sample) /liquid (deionized water) ratio was 1:10. Then, the solid and liquid were introduced into the rotating mixer with 30 r/min and 18 h. The leaching results of heavy metals (As, Cr, Zn, and Cu) in CMM and CFB fly ash were determined using high precision Thermofil ICP-MS (RQ type) equipment. Finally, the results of heavy metal leaching were compared with WHO International standards for safe drinking water.

## 3. Results and Discussion

### 3.1. Mechanical Properties

**Figure 5:**
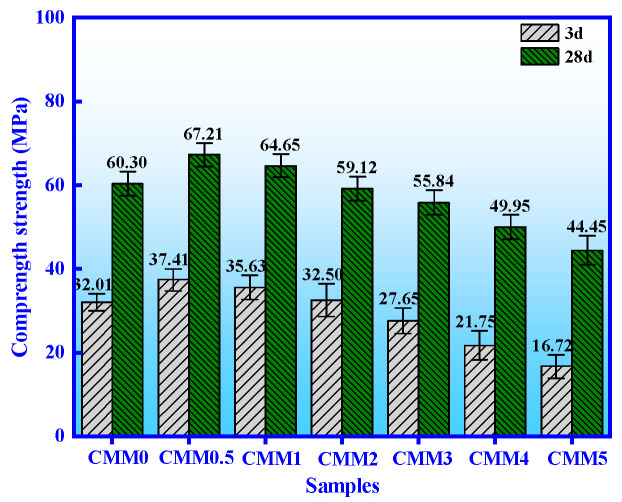
Compressive strength of the CMM cured for 3 to 28 days.

Figure 5 shows the compressive strength of CMM0~CMM5 at 3 and 28 days. The dispersion coefficient range of CMM is lower than 0.2, which indicates that these experimental values are close to the average. It is obvious that the compressive strength of CMM first increases and then decreases with the mass ratio of Ca/(Si+Al) from 2.38 to 0.83. The compressive strength of CMM is at its maximum value when the Ca/(Si+Al) was 2.13, which was 37.41 and 67.21 MPa at 3 and 28 days, respectively. Therefore, the strength value of CMM0.5 meets the 52.5 OPC strength standard in GB/T17671-1999 [27]. The reason for this phenomenon is that the CaSO_4_ (a mineral containing SO_3_) and active silica-alumina compositions in CFB fly ash are more beneficial to the generation of secondary hydration products of CMM0.5 [1]. Thereinto, the ettringite was synthesized by the early hydration reaction between CaSO_4_ in CFB fly ash and calcium aluminate in OPC. CMM0.5~5 contain ettringite because CFB fly ash contains SO_3_. The optimum strength at 3 days was presented at CMM0.5 based on the synergistic of ettringite and tricalcium silicate hydrate. Alongside this, the secondary hydration reaction of the active silica–alumina and the Ca(OH)_2_ produced calcium silica–aluminate gel (C-A-S-H gel) [31]. The compressive strength of CMM0.5 was increased at 28 days. Therefore, the mechanical properties of CMM0.5 are optimal, and they are composed of 5 wt.% CFB fly ash and 95 wt.% OPC.

**Figure 6:**
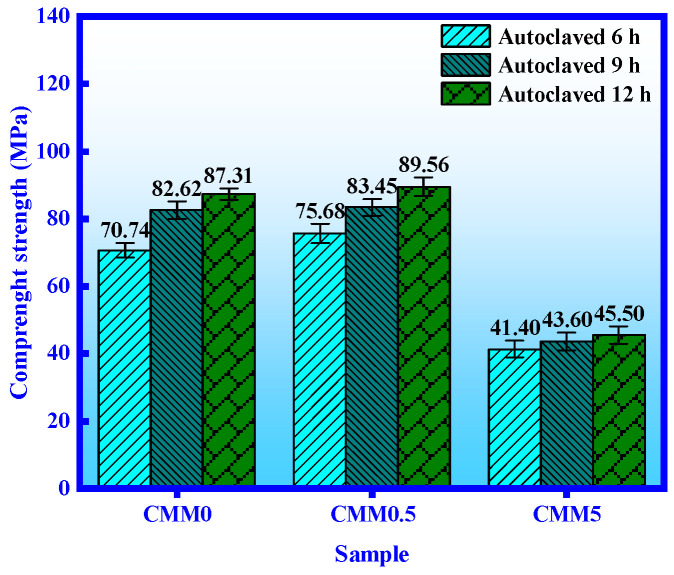
Compressive strength of CMM for autoclaved at 6, 9 and 12 h.

The CMM0, CMM0.5 and CMM5 were selected to investigate compressive strength of CMM mortar under autoclaved curing condition. As shown in Figure 6, the compressive strength of CMM mortar increases with the autoclaved time. The dispersion coefficients of CMM0, CMM0.5 and CMM5 are lower than 0.15 based on standard deviation calculation. This phenomenon shows that the experimental value of autoclaved strength is closer to the mean value. The compressive strength of CMM0–12 h (OPC) mortar is 87.31 MPa, which is higher than that of standard curing for 360 days (70 MPa) [32]. This result indicates that the hydration reaction rate of OPC is raised by autoclaving relative to standard curing. The compressive strength of CMM showed an inverted V-shape with the Ca/(Si+Al) mass ratio from 2.38 to 0.83, and the compressive strength of CMM0.5 was the highest. This proves that the hydration degree of CMM0.5 under autoclaved condition is higher based on the synergy of CFB fly ash and OPC. These rules are consistent with Figure 5.

### 3.2. Hydration Heat

**Figure 7:**
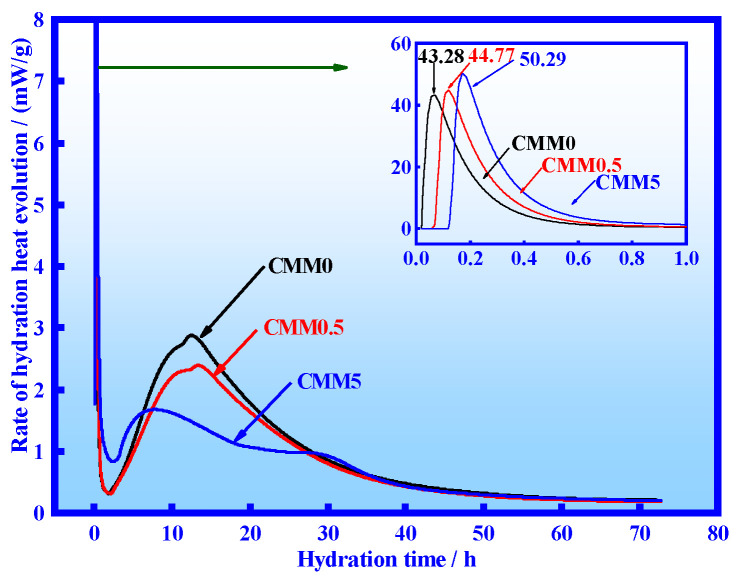
CMM hydration heat evolution rate within 72 h.

**Figure 8:**
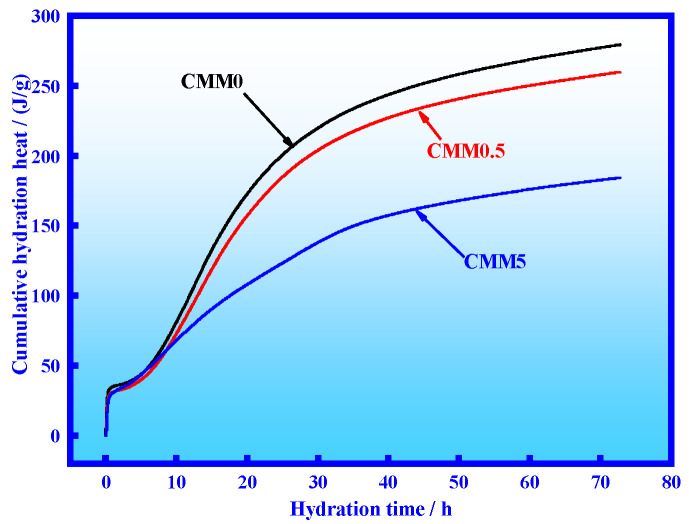
CMM cumulative hydration heat within 72 h.

The influence of CFB fly ash on the hydration of CMM was analyzed via isothermal calorimetry. The exothermic rates of CMM0, CMM0.5 and CMM5 are shown in Figure 7, and the cumulative hydration heats are shown in Figure 8. It is apparent that the peak value of CMM0~5 exothermic rate within 1h gradually increased from 43.28 to 50.29 mW/g. The first exothermic peak is formed in a short time because calcium aluminate reacts rapidly with CaSO_4_ to form ettringite and release surface energy [33,34]. The second exothermic peak (main exothermic peaks) of CMM appeared at 9~14 h because of rapid hydration reaction of Ca_3_SiO_5_(C_3_S) in a cement clinker [33]. The main exothermic rate of CMM0, 0.5 and 5 decreased gradually with the reduction of cement clinker content in CMM. The third exothermic peak of CMM5 appears at 30 h, which is supposed to be caused by the reaction of CFB fly ash and Ca(OH)_2_ to form C-A-S-H gels.

From the data in Figure 8, it is apparent that the cumulative hydration heat of CMM gradually decreased with the increase of CFB fly ash content at the same time. The cumulative hydration heat values of CMM0.5 and CMM5 at 72 h were 259.85 and 184.31 J/g, which decreased by 6.96% and 36.55% compared with CMM0, respectively. This result illustrates that the cumulative hydration heat of CMM is reduced by adding CFB fly ash. 

### 3.3. Volume Stability

**Figure 9:**
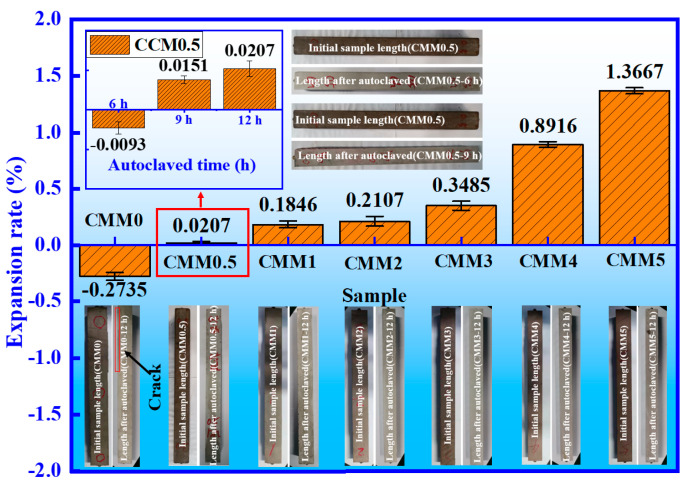
The expansion rates of CMM-12 h and CMM0.5 at different autoclaved times.

The volume shrinkage of cement leads to cracking in late hydration [35]. Furthermore, magnetization, freeze-thaw, harmful ion reactions and reinforcement corrosion hazards are easily caused due to cement cracking [36]. Therefore, volume stability is an important index of cement durability. The expansion rates of CMM0~CMM5 at 12 h and CMM0.5 at 6, 9 and 12 h are provided in Figure 9. The parameters related to CMM expansion rate in the autoclaved experiment are provided in Table 4. The dispersion coefficient of CMM expansion rate is lower than 0.11, which indicates that the experimental value of the expansion rate is approximately equivalent to the average. The f-CaO increased from 0 to 2.29 wt.% as the Ca/(Si+Al) mass ratio from 2.38 to 0.83. At the same time, the expansion rate of the CMM-12 h increased gradually from −0.2735 to 1.3667%. These results suggest that the f-CaO reacts with water to form Ca(OH)_2_, which leads to the volume expansion of CMM-12 h. The corresponding chemical reaction equation is shown in Equation (2).

(2) $${\mathrm{f-}\mathrm{CaO}+H}_{2}{O=\mathrm{Ca}(\mathrm{OH})}_{2}$$

Among them, the expansion rate of CMM0-12 h is −0.2735% (the crack is circled in red in Figure 9) because of thermal expansion and cold contraction of cement hydration. This result is consistent with the results of Shi [35]. The expansion rates of CMM4–12 h and CMM5-12 h are 0.8916% and 1.3667%, respectively, which are higher than the standard value (0.5%) of GB/T 750-1992. However, the expansion rate of the CMM0.5–12 h is the lowest (0.0207%). This phenomenon proves that the long-term volume stability of CMM0.5 is optimal compared to the others. In Figure 9, autoclaved experiments of CMM0.5 were conducted at 6, 9 and 12 h to explore the rule of CMM0.5 expansion rate. It is apparent that the expansion rate of CMM0.5 increases from −0.0093 to 0.0207% with the autoclave time. This phenomenon proves that the higher strength (Figure 5) contributes to the long-time volume stability of CMM0.5.

**Figure 10:**
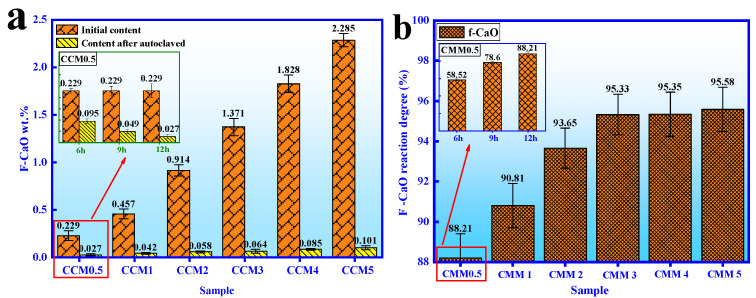
The f-CaO content (**a**) and f-CaO reaction degree (**b**) of CMM.

The content of f-CaO in CMM was detected according to the YB/T 4328–2012 [29] to quantitatively analyze the chemical reaction degree of f-CaO. The content of Ca(OH)_2_ was calculated using thermogravimetric results. Then, the mass fraction of f-CaO was obtained by subtracting Ca(OH)_2_ from total calcium (f-CaO + Ca(OH)_2_). Figure 10a shows the f-CaO content of CMM-12h and CMM0.5 at 6, 9 and 12 h. The initial content of f-CaO in the CMM increased from 0.299 to 2.285 wt.% as the Ca/(Si+Al) mass ratio decreased from 2.13 to 0.83. However, the content of f-CaO in all samples was lower than 0.11wt.% after autoclaved curing for 12 h. It is apparent from Figure 10b that the reaction degrees of f-CaO in CMM0.5~CMM5 at autoclaved 12 h were 88.21%, 90.81%, 93.65%, 95.33%, 95.35% and 95.58%, respectively. These results indicate that it is beneficial for the hydration of f-CaO to generate Ca(OH)_2_ with the decrease of Ca/(Si+Al) mass ratio. This also explains why the expansion rate of CMM-12h increases as the Ca/(Si+Al) mass ratio decreases from 2.38 to 0.83.

The initial content of f-CaO in CMM0.5 was 0.229%, while the content decreased from 0.096% to 0.027% after autoclave from 6 h to 12 h. As shown in Figure 10b, the reaction degree of f-CaO in CMM0.5 at 6, 9 and 12 h of autoclave was 58.52%, 78.60% and 88.21%, respectively. This result proves that the hydration reaction of f-CaO in CMM0.5 contributes to the formation of Ca(OH)_2_ with the autoclaved time. This also explains that the expansion rate of CMM0.5 gradually increases with the autoclaved time.

### 3.4. Autoclaved Hydration Characteristics

#### 3.4.1. XRD

**Figure 11:**
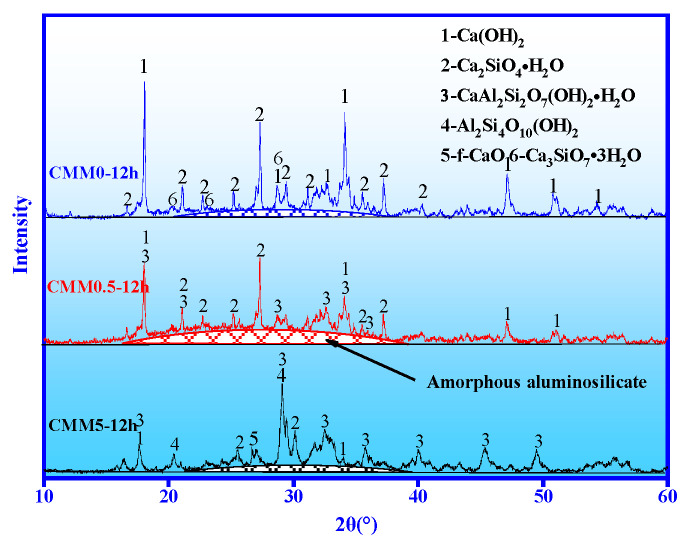
XRD results of CMM0, CMM0.5 and CMM5 at 12 h.

Three representative samples of CMM were examined using XRD to investigate the mechanism of compensate for shrinkage. Figure 11 shows the XRD results of CMM0, CMM0.5 and CMM5 under autoclave for 12 h. The results in Figure 11 indicate that the diffraction peak intensity corresponding to Ca(OH)_2_ is the highest, which is the sum of f-CaO and OPC hydration. Alongside this, the diffraction peak intensity of Ca(OH)_2_ decreases gradually with the Ca/(Si+Al) mass ratio from 2.38 to 0.83.

From Figure 9, Figure 10 and Figure 11, these results suggest that the proportion of Ca(OH)_2_ generated by f-CaO increases gradually and also indicate the gradual increase in the expansion rate from CMM0 to CMM5. The diffraction peak intensity of C-S-H, C-A-S-H and amorphous aluminosilicate shows an inverted V-shape change with the mass ratio of Ca/(Si+Al) from 2.38 to 0.83. The diffraction peak of minerals in CMM0.5 is the highest. Firstly, silicate of OPC is hydrated to form C-S-H gel and Ca(OH)_2_ (Equations (3) and (4). Secondly, the activated silica and aluminum in CFB fly ash are activated by Ca(OH)_2_ to form C-A-S-H gel. This can explain the chemical bond breaking and recombination of Al-O and Si-O, and the subsequent combination with Ca^2+^ to produce a new product: C-A-S-H gel (Equations (5)–(7)). The chemical reaction equation can be shown as below:

(3) $${3\mathrm{CaO}•\mathrm{SiO}}_{2}{+nH}_{2}{O=x\mathrm{CaO}•\mathrm{SiO}}_{2}{•yH}_{2}{O\;(C\mathrm{-S}\mathrm{-H})+(3-x)\mathrm{Ca}(\mathrm{OH})}_{2}$$

(4) $${2\mathrm{CaO}•\mathrm{SiO}}_{2}{+nH}_{2}{O=x\mathrm{CaO}•\mathrm{SiO}}_{2}{•yH}_{2}{O\;(C\mathrm{-S}\mathrm{-H})+(2-x)\mathrm{Ca}(\mathrm{OH})}_{2}$$

(5) $${\mathrm{SiO}}_{2}{+\mathrm{OH}}^{-}{+H}_{2}O\to {{[H}_{3}{\mathrm{SiO}}_{4}]}^{-}$$

(6) $${\mathrm{AlO}}_{2}{}^{-}{+\mathrm{OH}}^{-}{+H}_{2}O\to {{[H}_{3}{\mathrm{AlO}}_{4}]}^{2-}$$

(7) $${{[H}_{3}{\mathrm{SiO}}_{4}]}^{-}{+[H}_{3}{\mathrm{AlO}}_{4}{]}^{2-}{+\mathrm{Ca}}^{2+}\to C\mathrm{-A}\mathrm{-S}\mathrm{-H}$$

However, ettringite has not been found because it decomposes at 70 °C [37], and it was known that the expansion of CMM was mainly attributed to f-CaO. Therefore, the volume stability and compressive strength of CMM0.5–12 h are optimal based on the synergy of CFB fly ash and OPC.

**Figure 12:**
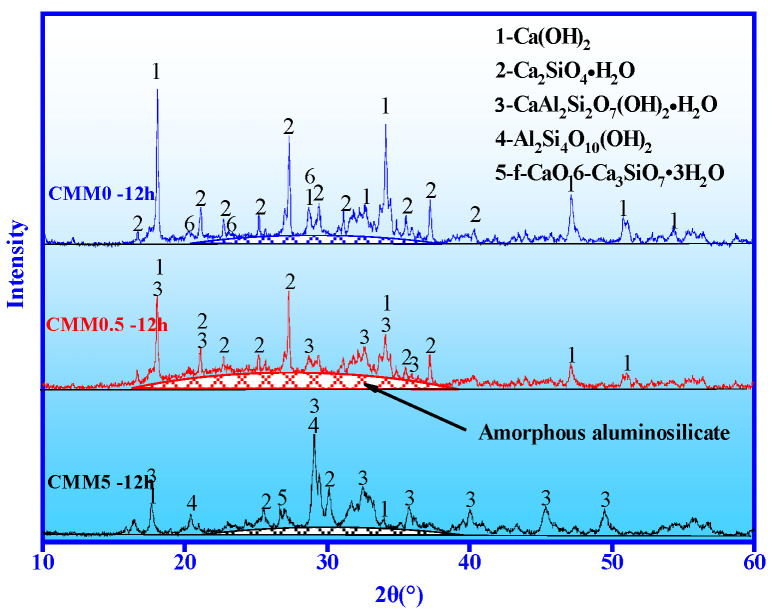
XRD results of CMM0.5 at 6, 9 and 12 h.

Autoclaved tests were conducted on CMM0.5 at 6, 9 and 12 h, and the mechanism of compensate for shrinkage in CMM0.5 with autoclaved time was investigated. It is apparent from Figure 12 that the diffraction peak intensity of Ca(OH)_2_ decreases gradually with the autoclaved time. Combined with the analysis in Figure 9, Figure 10, Figure 11 and Figure 12, it is obvious that the proportion of the Ca(OH)_2_ produced from the reaction of f-CaO gradually increases. This is why the expansion rate of CMM0.5 increases gradually with the autoclaved time. In addition, the diffraction peak strength of C-A-S-H gel and amorphous aluminosilicate increased gradually with the autoclaved time. Therefore, the expansion rate and compressive strength of CMM0.5 gradually increased with the autoclaved time.

#### 3.4.2. SEM-EDX

**Figure 13:**
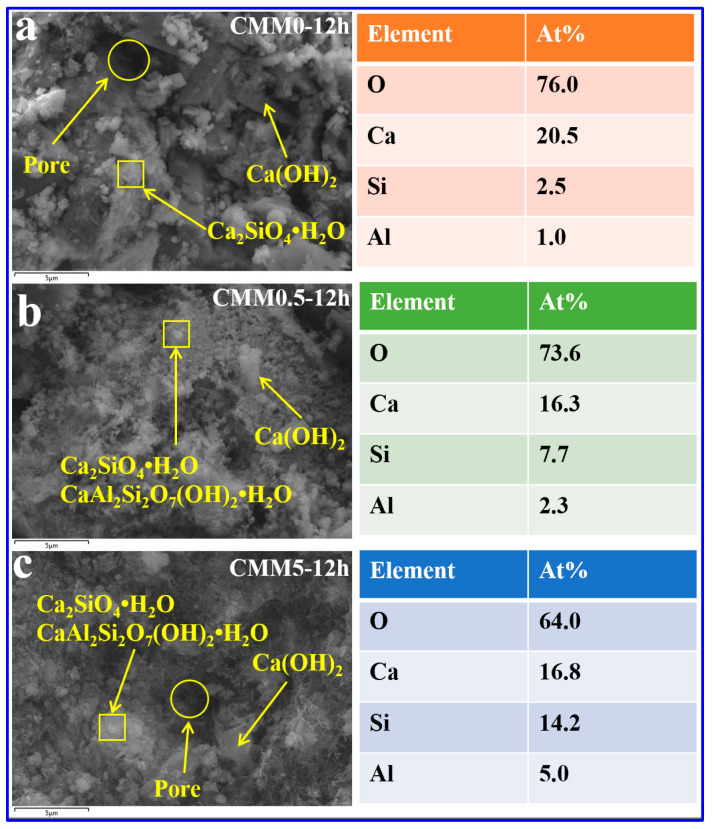
SEM-EDX results of (**a**) CMM0, (**b**) CMM0.5 and (**c**) CMM5 at autoclaved 12 h.

Three representative samples CMM0, CMM0.5 and CMM5 were selected to reveal the compensating shrinkage mechanism via microstructure analysis. The microscopic morphology and mineral distribution of CMM0–12 h, CMM0.5–12 h and CMM5–12 h are provided in Figure 13. It is apparent from Figure 13a that the overall microstructure of CMM0–12 h is loose and porous. Many flocculent structures are C-S-H gels, and the plate-like structure of Ca(OH)_2_ is exposed around C-S-H gel matrix [38]. It can be explained that the volume shrinkage of cement in the late hydration leads to its microstructure defects. In Figure 13b, CMM0.5–12 h showed a uniform microstructure compared with CMM0–12 h. In the meantime, many C-S-H, C-A-S-H gel and plate-like Ca(OH)_2_ agglomerated together with a densification structure. The reason for the morphology is that the secondary reaction of active silicon aluminum in CFB fly ash with Ca(OH)_2_ produces many C-A-S-H gel products. Ca(OH)_2_ was produced by reaction of f-CaO with water, causing volume expansion to compensate for volume shrinkage of OPC. Therefore, the compressive strength and volume stability of CMM0.5 is optimal. It is apparent in Figure 13c that the main microstructure of CMM5 is composed by the plate-like Ca(OH)_2_ with some small unreacted particles. This phenomenon proved that the flocculated structures of C-S-H and C-A-S-H gel are destroyed. Many pores were appeared due to the volume over-expansion of f-CaO. Therefore, these results of SEM-EDX are consistent with those in Figure 11 (XRD).

**Figure 14:**
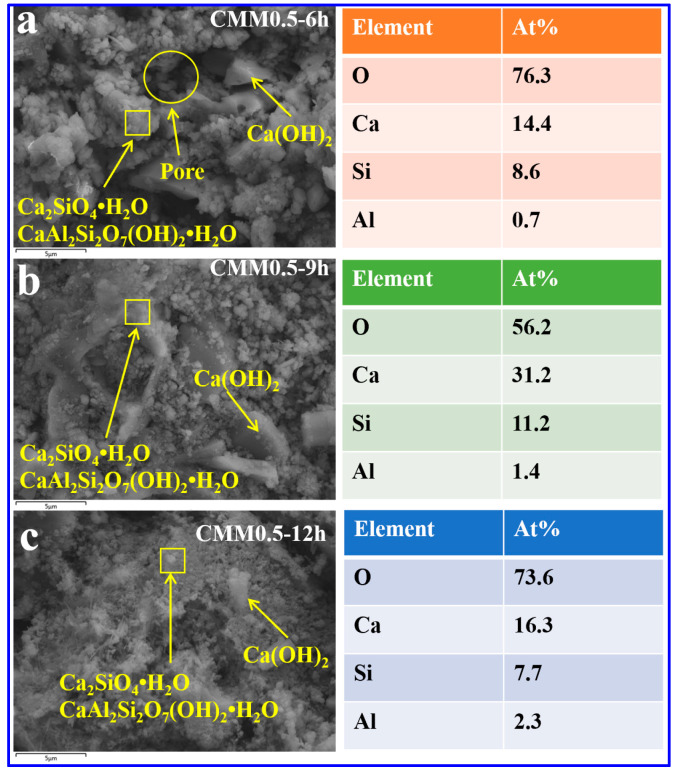
SEM-EDX results of CMM0.5 at autoclaved (**a**) 6, (**b**) 9 and (**c**) 12 h.

The mechanism of compensate for shrinkage of CMM0.5 were discussed with the autoclaved time. The micrographs and local element distribution of CMM0.5 autoclaved for 6, 9 and 12 h are shown in Figure 14a–c. The proportion of C-S-H and C-A-S-H gel in CMM0.5 gradually increased, while the pores gradually decreased with the autoclavic time. Therefore, CMM0.5–12 h exhibits a compact microstructure. This phenomenon indicates that the formation of C-S-H and C-A-S-H gels in CMM0.5 was promoted with the extension of autoclaved time. Meanwhile, the particle size of the plate-like Ca(OH)_2_ gradually increased with the autoclave time. The Ca(OH)_2_ in CMM0.5 was uniformly cohesive with the flocculating gel product when the autoclaved time was 12 h. According to the analysis of Figure 10 and Figure 14, the hydration reaction of f-CaO was promoted with the extension of autoclaved time. Thus, the compressive strength and volume stability of CMM0.5 gradually increase with autoclaved time. Therefore, these SEM-EDX results correspond to the XRD results in Figure 12.

#### 3.4.3. ^29^Si MAS NMR

Nuclear magnetic resonance spectroscopy (NMR) is the most effective tool for qualitative and quantitative analysis of components and molecular structures of various inorganic materials. The coordination structure of silicon was calculated by analyzing the chemical shift of ^29^Si resonance spectrum [39]. The number of adjacent coordination bridging oxygen atoms of ^29^Si is denoted by SiQ*^n^* (*n* = 0–4 integer). Zhang [40,41] found that the polymerization degree of silicon oxygen molecular structure was expressed by the relative bridging oxygen amount (RBO). Therefore, the polymerization degree of silicon oxygen was calculated by different coordination bridge oxygen numbers. The specific calculation formula of RBO value is as follows:

(8) $$\mathrm{RBO}=\frac{1}{4}(1\;\times \;\frac{Q^{1}}{\sum Q^{n}}+2\;\times \;\frac{Q^{2}}{\sum Q^{n}}+3\;\times \;\frac{Q^{3}}{\sum Q^{n}}+4\;\times \;\frac{Q^{4}}{\sum Q^{n}})=\frac{1}{4}\frac{\sum n\cdot Q^{n}}{\sum Q^{n}}$$

where Q*^n^* is the peak area of Si with *n* oxygen number in the coordination bridge.

**Figure 15:**
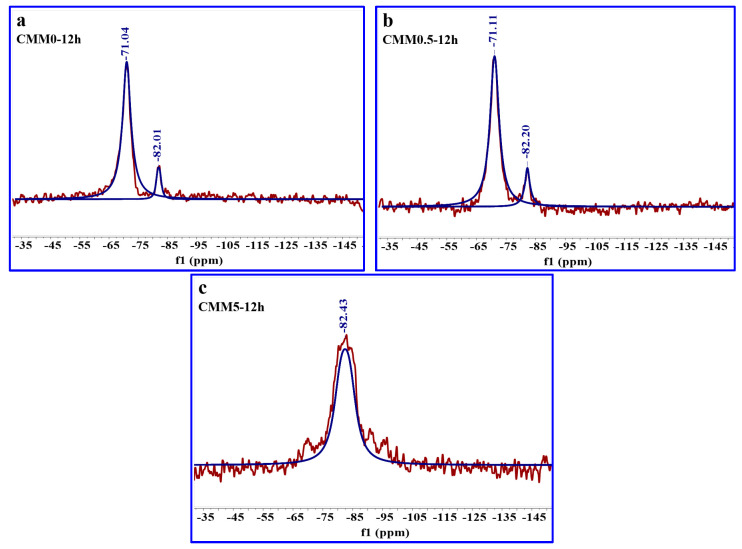
^29^Si NMR spectra of (**a**) CMM0, (**b**) CMM0.5 and (**c**) CMM5 samples autoclaved for 12 h.

As shown in Figure 15 and Table 5, the ^29^Si NMR spectrogram and RBO values of CMM0–12 h, CMM0.5–12 h, and CMM5–12 h were provided. The hydration products are mainly divided into five molecular structures: SiQ^0^, SiQ^1^, SiQ^2^(1Al), SiQ^3^(2Al) and SiQ^4^. The resonance spectra of SiQ*^n^* were fitted with MestReNova software, and then the relative peak area of each SiQ*^n^* was obtained. Finally, the RBO value is calculated by Equation (8), and these results are provided in Table 5 and Table 6. The molecular structure of SiQ^0^ is the unreacted Ca_2_SiO_4_ or Ca_3_SiO_5_ in CMM, and the molecular structure of other SiQ*^n^* is related to C-S-H gel and C-A-S-H gel [42]. In ^29^Si NMR spectrum, SiQ^2^(1Al) is part of silicon atoms in [SiO_4_] replaced by aluminum atoms to form a new [Si(Al)O_4_]. This [Si(Al)O_4_] molecular structure corresponds to the formation of C-A-S-H gel. From the data in Table 5 and Table 6, SiQ^2^(1Al) presents a certain peak area in CMM, which indicates that C-A-S-H gel exists in CMM. Hence, more C-A-S-H gel phases are found in the XRD pattern. The relative peak area of SiQ^2^(1Al) gradually increased from 10.84 to 100 with the mass ratio of Ca/(Si+Al) from 2.38 to 0.83, while the SiQ^1^ peak of CMM5 disappeared. Therefore, the number of hydration products corresponding to CMM0.5 is the largest. In addition, it is clear that the RBO value changes in an inverted V-shape with the Ca/(Si+Al) mass ratio from 2.38 to 0.83. The RBO value is the highest (28.35%) when the Ca/(Si+Al) mass ratio is 2.13. In summary, these results suggest that the degree of CMM0.5 polymerization is optimal.

**Figure 16:**
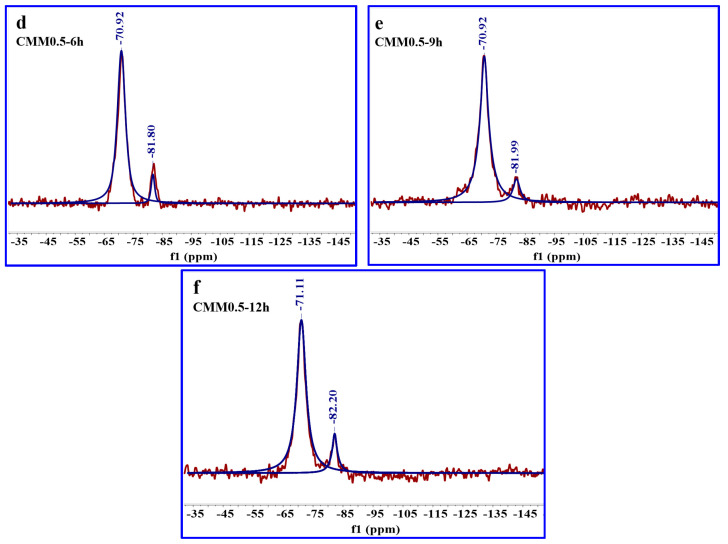
^29^Si NMR spectra of CMM0.5 autoclaved for (**d**) 6, (**e**) 9 and (**f**) 12 h.

As shown in Figure 16 and Table 6, the ^29^Si NMR spectrograms and RBO values of the CMM0.5–6 h, CMM0.5–9 h, and CMM0.5–12 h were supplied. It is clearly seen that the relative peak area of SiQ^2^ gradually increases from 8.01 to 15.46 with the autoclaved time. This phenomenon indicates that CMM0.5 is beneficial to the synthesis of [SiO_4_] and [Si(Al)O_4_] with autoclaved time, which generates more C-S-H and C-A-S-H gel. It is apparent from Table 6 that the RBO value gradually increases with the autoclaved time. The maximum degree of polymerization of CMM0.5 is 28.35% when the autoclaved time is 12 h. This result illustrates that the polymerization degree of [SiO_4_] and [Si(Al)O_4_] at CMM0.5 is improved by prolonging the autoclaved time. In summary, mechanical properties and volume stability of CMM0.5 are optimal because the polymerization structure is ameliorated through the molecular polymerization degree of CMM0.5 [40]. The mechanical properties and volume stability of CMM0.5 are enhanced gradually with the autoclaved time. Therefore, the ^29^Si NMR results of CMM is consistent with the XRD and SEM-EDX results.

### 3.5. Environmental Performance

#### 3.5.1. Heavy Metal Leaching

The leaching of the heavy metals in CFB fly ash could pollute groundwater and endanger human health. The environmental performance of the CFB fly ash materials should be evaluated to meet environmental requirements. According to GB 5086.1-1997 standard [30], heavy metals leaching of CFB fly ash, CMM0.5 and CMM5 were examined. The leaching concentration of heavy metals was determined using an ICP-MS instrument, which was compared with the WHO for drinking water [42,43]. Heavy metal leaching results are shown in Table 7.

In Table 7, the concentrations of As and Cr in CFB fly ash are 0.32175 and 0.18767 mg/L, respectively, and these results exceed the requirements of WHO drinking water standards. However, the concentrations of Zn and Cu are 0.84240 mg/L and 0.14650 mg/L, respectively, which meet the WHO standards for drinking water. In addition, the concentrations of heavy metals in CMM0.5 and CMM5 meet the WHO standards for quality concentration limits of drinking water. These results suggest that the heavy metals of CFB fly ash are effectively consolidated by CMM. Interestingly, the heavy metal leaching concentration of CMM0.5 is much lower than that of CMM5, which indicates that CMM0.5 is an environment-friendly cementitious material.

#### 3.5.2. EPMA

**Figure 17:**
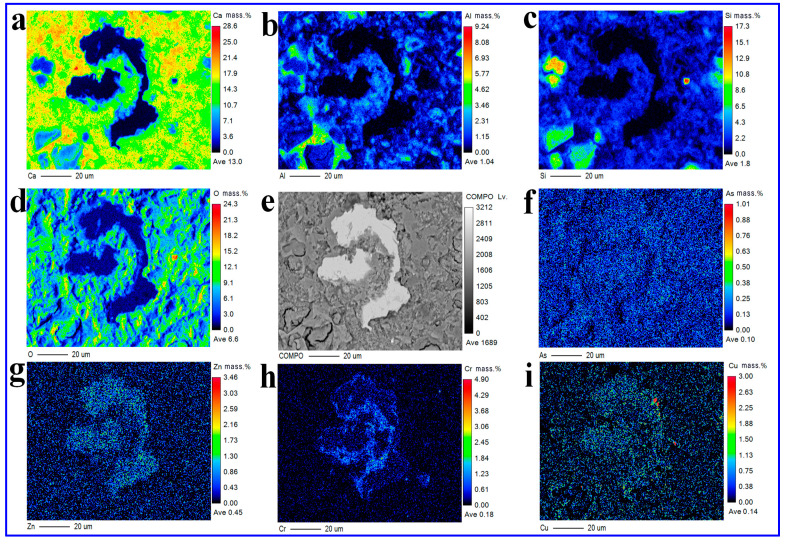
BSE image and elemental distribution of CMM0.5–12 h (**a**–**i**).

EPMA is a common detection method for element distribution and quantitative analysis in CMM [44]. The CMM paste was embedded in resin (20 mm in diameter, 10 mm in height). Then, the surface was polished. The polished surface of CMM0.5–12 h was tested using the EPMA instrument. Figure 17 shows the BSE image of CMM0.5–12 h paste and the distribution of Ca, Al, Si, O, As, Cr, Zn and Cu. It is clearly seen that Ca, Si and O are the main elements of CMM0.5–12 h corresponding to the C-S-H gel phase, which is consistent with XRD, SEM-EDS and ^29^Si MAS results. As shown in Figure 17b, the content of Al is between 0∼9.24 wt.% (the average content is 1.04 wt.%), and the layout is consistent with the distribution of Ca and Si. These results are consistent with those of C-A-S-H gel found by XRD, SEM-EDS and ^29^Si MAS.

As shown in Figure 17f,h, the average content of As is 0.10 wt.%, and it is evenly distributed in the CMM0.5. Meanwhile, average content of Cr is 0.18 wt.%, which is mainly distributed in the non-enriched area of Ca, Al, Si and O. This was consistent with the results of Liu [44]; that is, Cr was absorbed by C-S-H gel. Si is replaced by As in the C-S-H gel, or As is adsorbed by C-S-H gel to generate hydration products containing As element. As shown in Figure 17i, the average content of Cu is 0.14 wt.%. Some Cu elements are concentrated in Ca, Al, Si and O regions. As shown in Figure 17g, the average content of Zn is 0.45 wt.%, and its distribution was similar to that of Cu. These phenomena indicates that Cu and Zn are probably adsorbed by C-S-H gel or participates in new chemical reactions to form minerals [45].

#### 3.5.3. Consolidation Mechanism

**Figure 18:**
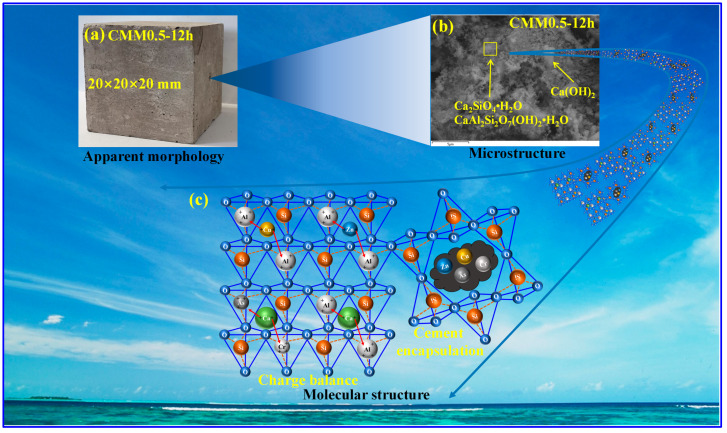
The consolidation mechanism of heavy metals of the CMM0.5–12 h ((**a**): Apparent; (**b**): Microstructure; (**c**): Molecular).

The possible consolidation mechanism of heavy metals in CMM0.5–12 h is shown in Figure 18. Figure 18a is the apparent morphology of the CMM0.5–12 h, and the whole surface of CMM0.5–12 h is smooth with microholes. Figure 18b is the microstructure of the CMM0.5–12 h, the main microstructure is flocculent C-S-H, C-A-S-H gel and a small amount of Ca(OH)_2_. Figure 18c is molecular structure of the CMM0.5–12 h. Minerals containing heavy metals are encapsulated by molecular rings consisting of [SiO_4_] tetrahedra in cement. However, the charge balance is that Si^4+^ of [SiO_4_] is replaced by Al^3+^ to form [Si(Al)O_4_] with a negative charge [46]. Then, Cu^2+^ or Zn^2+^ is consolidated by the two [Si(Al)O_4_] molecular structures to achieve the charge balance. In addition, another possible explanation for this is that As and Cr are isomorphic to form [Si(As)O_4_] and [Si(Cr)O_4_] structures with a negative charge based on the [SiO_4_] coordination isomorphic. Ca^2+^ is combined with [Si(As)O_4_] or [Si(Cr)O_4_] to achieve a stable structure. Therefore, the consolidation mechanism of heavy metals are the synergy of cement encapsulation and charge balance [42].

## 4. Conclusions

The mechanical properties, volume stability, f-CaO shrinkage compensation, and environment friendliness of CMM are studied in this work. These conclusions are as follows:

(1) The normal and autoclaved strength of CMM0.5 are the highest. The compressive strength of CMM0.5 autoclaved for 6, 9 and 12 h is 75.68 MPa, 83.45 MPa and 89.56 MPa, respectively. In addition, the compressive strength of CMM0.5 is 37.41 MPa and 67.21 MPa at normal curing 3 and 28 days, which satisfies the standard of 52.5 OPC.
(2) CMM0.5 shows the lowest expansion rate (0.0207%), higher strength, and lower hydration heat. The long-term volume stability of CMM0.5 satisfies the cement expansion standard (≤0.5%) and avoids cement cracking.
(3) The main autoclaved hydration products of CMM0.5 are C-S-H, C-A-S-H gel and Ca(OH)_2_. The densified microstructure and high polymerization degree are presented at CMM0.5. The microstructure and polymerization degree of the hydration product are beneficial to the strength development and long-term volume stability of CMM0.5.
(4) Heavy metals are effectively consolidated by CMM0.5 due to physical encapsulation and charge balance. The lowest leaching concentration of heavy metals is shown at CMM0.5, which meet the WHO standards for drinking water. Therefore, CMM0.5 is an environment-friendly cementitious material with long-term volume stability.

## Figures and Tables

**Table 1:** Chemical components of CFB fly ash and OPC (wt.%). Note: LOI: Loss of ignition at 800 °C.

Oxide	T-CaO	SiO_2_	Al_2_O_3_	SO_3_	Fe_2_O_3_	MgO	TiO_2_	K_2_O	Na_2_O	f-CaO	LOI
CFB fly ash	14.87	39.90	28.09	7.73	6.21	1.46	0.98	0.95	0.23	4.57	10.38
OPC	63.15	21.05	5.43	2.66	3.56	1.86	0.38	0.65	0.27	-	3.42

**Table 2:** Designed proportion of different CMM.

Mix Sample	CFB Fly Ash (wt.%)	CFB Fly Ash (kg/m^3^)	OPC (kg/m^3^)
CMM0	0	0	450
CMM 0.5	5.0	22.5	427.5
CMM 1	10	45	405
CMM 2	20	90	360
CMM 3	30	135	315
CMM 4	40	180	270
CMM 5	50	225	225

**Table 3:** Chemical components of CMM (mass fraction/%).

Oxides	CMM0	CMM0.5	CMM1	CMM2	CMM3	CMM4	CMM5
T-CaO	63.15	60.74	58.32	53.49	48.67	43.84	39.01
f-CaO	0	0.23	0.46	0.91	1.37	1.83	2.29
SiO_2_	21.05	21.99	22.94	24.82	26.71	28.59	30.48
Al_2_O_3_	5.43	6.56	7.70	9.96	12.23	14.49	16.76
SO_3_	2.66	2.91	3.17	3.67	4.18	4.69	5.20
Fe_2_O_3_	3.56	3.69	3.83	4.09	4.36	4.62	4.89
MgO	1.86	1.84	1.82	1.78	1.74	1.70	1.66
TiO_2_	0.38	0.41	0.44	0.50	0.56	0.62	0.68
K_2_O	0.65	0.67	0.68	0.71	0.74	0.77	0.80
Na_2_O	0.27	0.27	0.27	0.26	0.26	0.25	0.25
Ca/(Si+Al) mass ratio	2.38	2.13	1.90	1.54	1.25	1.02	0.83

**Table 4:** CMM autoclaved test parameters.

Sample	Initial Length (mm)	After Autoclaved (mm)	Length Change (mm)	Expansion Rate (%)
CMM0.5–6 h	296.4345	296.4070	−0.0275	−0.0093%
CMM0.5–9 h	297.2860	297.3310	0.0450	0.0151%
CMM0.5–12 h	296.9880	297.0495	0.06150	0.0207%
CMM0–12 h	296.9040	296.0920	−0.8120	−0.2735%
CMM0.5–12 h	296.9880	297.0495	0.06150	0.0207%
CMM1–12 h	296.2830	296.8300	0.5470	0.1846%
CMM2–12 h	297.5940	298.2210	0.6270	0.2107%
CMM3–12 h	295.8320	296.8630	1.0310	0.3485%
CMM4–12 h	294.5420	297.1680	2.6260	0.8916%
CMM5–12 h	293.3320	297.3410	4.0090	1.3667%

**Table 5:** The RBO values of CMM0, CMM0.5 and CMM5 samples autoclaved for 12 h.

Sample	Peak Position (ppm)	Assign	Relative	RBO Value
CMM0–12 h	−71.04	SiQ^1^	100.00	27.44%
	−82.01	SiQ^2^(1Al)	10.84	
CMM0.5–12 h	−71.11	SiQ^1^	100.00	28.35%
	−82.20	SiQ^2^(1Al)	15.46	
CMM5–12 h	−82.43	SiQ^2^(1Al)	100.00	25.00%

**Table 6:** The RBO values of CMM0.5 samples autoclaved for 6, 9 and 12 h.

Sample	Peak Position (ppm)	Assign	Relative	RBO Value
CMM0.5–6 h	−70.92	SiQ^1^	100.00	26.85%
	−81.80	SiQ^2^(1Al)	8.01	
CMM0.5–9 h	−70.92	SiQ^1^	100.00	27.73%
	−81.99	SiQ^2^(1Al)	12.24	
CMM0.5–12 h	−71.11	SiQ^1^	100.00	28.35%
	−82.20	SiQ^2^(1Al)	15.46	

**Table 7:** Leaching results of heavy metals (mg/L).

Sample	As	Cr	Zn	Cu
CFB fly ash	0.32175	0.18767	0.84240	0.14650
CMM0.5	0.00005	0.00711	0.00352	0.00062
CMM5	0.00210	0.01906	0.00738	0.00438
WHO drinking water standard	0.01000	0.05000	3.00000	1.00000

## Data Availability

Data sharing is not applicable to this article.
